# Discrete Element Modeling of Intermetallic Matrix Composite Manufacturing by Powder Metallurgy

**DOI:** 10.3390/ma12020281

**Published:** 2019-01-16

**Authors:** Szymon Nosewicz, Jerzy Rojek, Marcin Chmielewski, Katarzyna Pietrzak

**Affiliations:** 1 Institute of Fundamental Technological Research Polish Academy of Sciences, Pawińskiego 5B, 02-106 Warsaw, Poland; snosewi@ippt.pan.pl (S.N.); katarzyna.pietrzak@itme.edu.pl (K.P.); 2 Institute of Electronic Materials Technology, Wólczyńska 133, 01-919 Warsaw, Poland; marcin.chmielewski@itme.edu.pl

**Keywords:** powder metallurgy, sintering, discrete element method, modeling, intermetallic matrix composites

## Abstract

This paper presents a numerical and experimental analysis of manufacturing of intermetallic ceramic composites by powder metallurgy techniques. The scope of the paper includes the formulation and development of an original numerical model of powder metallurgy of two-phase material within the framework of the discrete element method, simulations of powder metallurgy processes for different combinations of process parameters, and a verification of the numerical model based on own experimental results. Intermetallic-based composite NiAl–Al${}_{2}$O${}_{3}$ has been selected as representative material for experimental and numerical studies in this investigation. Special emphasis was given to the interactions between the intermetallic and ceramic particles by formulating the special model for adhesive contact bond. In order to properly represent a real microstructure of a two-phase sintered body, a discrete element specimen was generated using a special algorithm. Numerical validation showed the correct numerical representation of a sintered two-phase composite specimen. Finally, micromechanical analysis was performed to explain the macroscopic behavior of the sintered sample. The evolution of the coordination number, a number of equilibrium contacts, and the distribution of the cohesive neck size with respect to time are presented.

## 1. Introduction

Powder metallurgy (PM) is a technology commonly used for manufacturing metal, ceramic or composite materials applicable in many industrial branches. From a technological point a view, powder metallurgy is a process consisting of mixing granular materials, compacting them into a desired form, heating and sintering the compressed material in a controlled atmosphere, and finally, cooling to room temperature. There are several techniques of powder metallurgy. Pressure-assisted sintering, involving simulatanous powder compaction and sintering, is one of the most common PM techniques [1]. If the pressure is applied uniaxially, the process is referred to as hot pressing.

**Figure 1:**
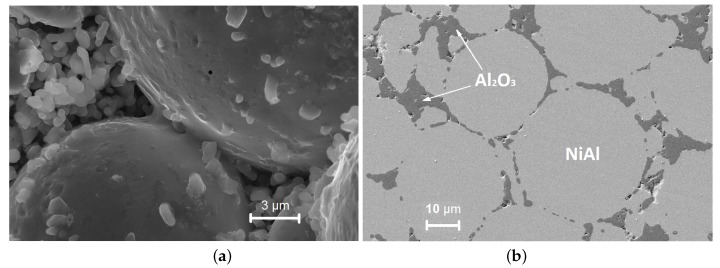
Structural change of the intermetallic NiAl–Al${}_{2}$O${}_{3}$ composite material during sintering at temperature $T_{s}=1400\;$C, sintering time $t_{s}=10$ min, and applied pressure p=30 MPa: (**a**) Before and (**b**) after final consolidation.

Sintering is the essential stage of a PM process consisting of the consolidation of a particulate material at high temperatures but below the melting point. As a result of the sintering process, a solid compact body is formed from the powder (Figure 1). The material microstructure during sintering undergoes changes due to particle compaction and rearrangement, generation and growth of cohesive bonds, leading to the reduction and elimination of porosity. The processes at the microscopic scale induces changes in the macroscopic physical properties.

Great progress in the technology of sintering and powder metallurgy techniques enables the permanent development of modern materials, such as composites—materials formed from two (or more) components (e.g., metallic, intermetallic or ceramic) with different physical and chemical properties, which together give different and usually improved characteristics with respect to individual components. In this work, attention is focused on the intermetallic matrix composite (IMC) reinforced with ceramics NiAl–Al${}_{2}$O${}_{3}$. Nickel aluminide based materials have numerous advantages, e.g., a high melting temperature, low density, high resistance to high-temperature oxidation (up to about 1200 C) [2,3], a high Young modulus and mechanical strength [4] or good resistance to abrasion. They have a significant potential in industrial applications [5], such as in jet engine hardware, energy conversion (i.e., stationary gas turbines of power plants), internal combustion engines, and heat exchangers [6,7,8].

The manufacturing of intermetallic NiAl-matrix composites is a complex and nontrivial issue [9]. Residual stresses and material cracking in composites are typical defects due to the differences in the atomic structure and properties between intermetallics and ceramic materials, but there are also other additional difficulties in the sintering of mixed powders in comparison to those encountered in the sintering of a single phase powder. Possible chemical interactions between phases, different sinterability, different particle sizes, sintering process parameters, including heating rate, sintering temperature, and time, are factors which should be carefully considered in the design of a sintering process for mixed powders [10].

Numerical modeling and simulations can help in understanding better the influence of powders’ characteristics and the process parameters on the manufacturing process and and final properties of the sintered material. Modeling of the powder metallurgy process of composites is quite a new and challenging research task. An efficient and effective model of powder metallurgy and sintering processes should allow us to accurately analyze the manufacturing process and evolution of the material during the process. It should predict the properties of the sintered material, such as its density, porosity, as well as its mechanical properties. Moreover, the possibility to assess residual stresses and the risk of cracking of the composite material during cooling is a desirable feature of a PM model.

Numerical modeling is a promising tool to study the complicated process of powder metallurgy of composites. However, the choice of an appropriate model is a fundamental issue. There are different approaches to modeling of powder metallurgy and sintering itself, including phenomenological and mechanistic approaches, continuous and discrete formulations, modeling at macro, micro, and atomistic levels, and multiscale modeling combining models at different levels. Different sintering models are reviewed in References [11,12,13,14].

Recently, a micromechanical particle approach of the sintering process was applied successfully, focusing on the microscopic scale of the material. Discrete models take into account the discontinuities, defects, molecular structure of the material, and its particle size. Discrete modeling has been developed in response to the deficiency of a continuous model associated with the inability to consider all kind of defects in the material, and the difficulties in formulating constitutive equations of those models. The discrete element method (DEM), which has been applied in this research, is based on a discrete element representation of the compacted powder, which is modeled by a large collection of rigid or deformable discrete elements interacting among one another with contact forces.

Generally, the contact interaction in DEM models of sintering is mostly based on the assumption that the process is governed by the viscous flow and neglects the elastic behavior [15,16,17]. Keeping in mind the importance of elastic effects for the proper evaluation of residual stresses, as well as for interparticle interaction, the viscous contact model used previously in many works has been extended by combining the spring and the viscous rheological element in series [18]. Consequently, the elastic and viscous effects in the particle interaction during sintering have been taken into account, which additionally ensured a much better efficiency. This model has been further enriched by the application of Hertz contact model interaction between the powder particles, which brought more realistic numerical results in comparison to experimental ones [19]. This formulation has been successfully employed to model a hot pressing process with its main stages: Initial powder compaction and pressure-assisted sintering within one simulation [20].

Mostly, discrete element models of the sintering process consider simulations of one-phase powder; only a few papers have explored the modeling of sintering of two-phase powders, e.g., [21,22,23]. Olmos et al. [23] analyzed the sintering of mixtures of copper and ceramic powders with different mixture compositions. The numerical studies were supported by experiments; however, the number of tests was quite small (density range from around 0.65 to 0.75) and the agreement between experimental and numerical results was not fully satisfactory. The influence of rigid inclusions on the sintering behavior of the matrix was investigated using DEM in Reference [21]. The model for the interaction between the matrix and rigid inclusion particles used in Reference [21] was developed by a generalization of the model proposed by Olmos et al. [23] for multisize particle mixtures. The densification behavior of metal–ceramic powder mixtures with varying phase compositions (0–100% metal content) was investigated numerically in Reference [22]. The analyses were performed at a constant temperature. The results were validated using the experimental results reported in Reference [21].

This paper presents a numerical modeling of a powder metallurgy (hot pressing) process of a two-phase powder mixture validated with our own experimental results. The original DEM model, which was applied in the simulation of the one-phase hot pressing process [20], is now extended to model a powder metallurgy process of an intermetallic composite specimen. Unlike other discrete element models of the two-phase powder mixture, which were only focused on the sintering process, our approach allows us to model the entire process of powder metallurgy with its subsequent steps, starting from the initial compaction of the powder by uniaxial loading, through subsequent consolidation during sintering, and finally, ending up with the cooling of the sintered material and unloading. The present study is one of the first efforts of discrete element modeling of two-phase sintering accounting for more than one type of interparticle interaction. Special emphasis has been placed on the interaction between the intermetallic and ceramic particles by formulating a special model for the adhesive contact bond.

The numerical investigation has been carried out for a powder metallurgy process performed to manufacture a composite with the NiAl intermetallic matrix reinforced with Al${}_{2}$O${}_{3}$ ceramics. The discrete element model has been generated using a special procedure which ensures a random spatial distribution of powder particles of each phase. The generation algorithm allows us to satisfy the main requirements of a real two-phase powder after mixing and compaction, such as the isotropy of composite material and the uniform distribution of reinforcement (ceramic) particles in the intermetallic NiAl matrix. This should also be highlighted in the context of the previously mentioned papers.

Own experimental results were been used to calibrate and validate the numerical model. The model was calibrated by fitting the numerical densification curve to the experimental data for a given set of process parameters. Finally, the calibrated model was validated by numerical simulations performed for different process parameters and by comparing the numerical and experimental results.

## 2. Discrete Element Model of the Powder Metallurgy Process

The numerical model of the powder metallurgy process for composite materials was developed within the framework of the discrete element method, with spherical discrete elements representing powder particles. Such a model explicitly takes into account the particulate nature of the sintered material.

The particles interact among one another with contact forces. The model of the contact interaction in the DEM plays the role of the microscopic constitutive model. This work is based on the viscoelastic contact model for powder sintering, developed bu the authors of [20] and enriched in Reference [19] by taking the Hertz model for the elastic contact. This model has been extended here to modeling two-phase powders, which equired a possibility to define specific interactions between particles of the same and different phases.

The powder metallurgy process is treated as a thermomechanical problem. The thermal problem is idealized by assuming a uniform temperature in the specimen. This is justified by the small sizes of the specimen considered in the analyzed examples. The temperature evolution in the whole specimen is prescribed so the heat conduction problem is not analyzed. One-way coupling between the thermal and mechanical problems was taken into account. The following thermal effects were taken into account in the solution of the mechanical problem:

- Thermal expansion of the particles and resulting thermal stresses;
- Effect of temperature on the diffusivity, which, in turn, influences viscous properties of the sintered material.

The developed model allows us to simulate all the stages of the PM process: Compaction, sintering, and cooling. The basic features of the interaction models for these stages are briefly reviewed.

### 2.1. Compaction

The interaction of powder particles at the compaction stage takes into account elastic deformation, viscous dissipation, and friction at the contact point. The normal contact is considered using the Kelvin–Voigt-type model. The normal contact force $F_{n}$ is composed of the nonlinear elastic force $F_{n}^{e}$ and the viscous component $F_{n}^{d}$:

(1) $$F_{n}=F_{n}^{e}+F_{n}^{d}=\frac{4}{3}\bar{E}\sqrt{\bar{r}}{u_{\mathrm{rn}}^{e}}^{\frac{3}{2}}+c_{n}v_{\mathrm{rn}},$$

where $\bar{E}$ is the effective Young’s modulus, $\bar{r}\left(T\right)$ is the effective radius of particle dependent on the temperature, $u_{\mathrm{rn}}^{e}$ is the particles penetration, $c_{n}$ is the coefficient of the viscosity, and $v_{\mathrm{rn}}$ is the normal relative velocity, $\bar{E}$ is the effective Young’s modulus:

(2) $$\frac{1}{\bar{E}}=\frac{1-\nu_{i}^{2}}{E_{i}}+\frac{1-\nu_{j}^{2}}{E_{j}},$$

$E_{a}$ and $\nu_{a}$, a=i,j, being the Young’s moduli and Poisson’s ratios of the contacting particles, respectively, and $\bar{r}$ is the effective radius:

(3) $$\frac{1}{\bar{r}}=\frac{1}{r_{i}}+\frac{1}{r_{j}},$$

$r_{a}$, a=i,j, being the contacting particles radii. The tangential contact force is evaluated assuming the regularized Coulomb friction model. Alternative contact models for powder compaction have been presented in Reference [24].

### 2.2. Sintering

The interaction model for the sintering stage takes into account elastic and inelastic (viscous) deformation. The viscoelastic contact model for sintering presented in Reference [19] has been used. The viscoelastic component in this model is represented by the Maxwell-type element composed of the Hertzian nonlinear spring connected in series with the dashpot element. As a consequence of the use of the Maxwell model, the relative normal velocity at the contact $v_{\mathrm{rn}}$ is decomposed into an elastic and viscous part, $v_{\mathrm{rn}}^{e}$ and $v_{\mathrm{rn}}^{v}$, respectively:

(4) $$v_{\mathrm{rn}}=v_{\mathrm{rn}}^{e}+v_{\mathrm{rn}}^{v}.$$

The forces transferred by the viscous and elastic elements, $F_{n}^{v}$ and $F_{n}^{e}$, respectively, are equal:

(5) $$F_{n}^{v}=F_{n}^{e}.$$

The interaction force can be written as the sum of the force in the Maxwell branch, $F_{n}^{v}$ or $F_{n}^{e}$, and the sintering driving force $F_{n}^{\mathrm{sint}}$:

(6) $$F_{n}=F_{n}^{\mathrm{sint}}+F_{n}^{v}=F_{n}^{\mathrm{sint}}+F_{n}^{e}.$$

The form of the elastic force is the same as the one shown in the compaction model (Equation (1)). The viscous force is given by:

(7) $$F_{n}^{v}=\eta v_{\mathrm{rn}}^{v},$$

where the viscosity coefficient η, following classical models of sintering developed at the particle level [25,26,27], can be expressed in terms of the effective grain boundary diffusion coefficient $D_{\mathrm{eff}}$:

(8) $$\eta =\frac{\pi a^{4}}{8D_{\mathrm{eff}}},$$

where *a* is the radius of cohesive neck. The effective grain boundary diffusion coefficient $D_{\mathrm{eff}}$ can be evaluated as:

(9) $$D_{\mathrm{eff}}=\frac{D_{\mathrm{gb}}\delta \Omega }{k_{B}T},$$

where Ω is the atomic volume, $k_{B}$ is the Boltzmann constant, *T* is the absolute temperature, and $D_{\mathrm{gb}}$ is the grain boundary diffusion coefficient with the width δ given by the Arrhenius-type equation [28]:

(10) $$D_{\mathrm{gb}}=D_{0\mathrm{gb}}\exp\left[-\frac{\Delta H_{\mathrm{gb}}}{RT}\right],$$

where $D_{0\mathrm{gb}}$ is the pre-exponential factor of grain boundary diffusion, $\Delta H_{\mathrm{gb}}$ is the activation enthalpy of grain boundary diffusion, and *R* is the gas constant.

**Figure 2:**
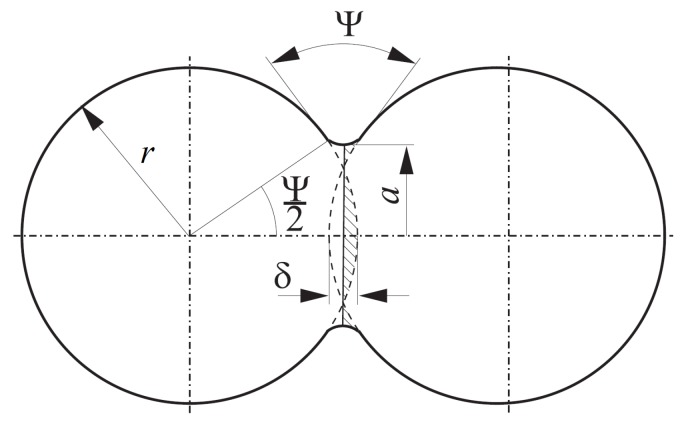
Two-particle model of sintering.

The sintering driving force $F_{n}^{\mathrm{sint}}$ results from surface tension on the particles grain boundary [15]:

(11) $$F_{n}^{\mathrm{sint}}=\pi \gamma_{S}\left[4\bar{r}\left(1-\cos\frac{\Psi }{2}\right)+a\sin\frac{\Psi }{2}\right],$$

where $\bar{r}$ is the effective particle radius, Ψ is the dihedral angle, $\gamma_{S}$ is the surface energy, and $a_{0}$ is the neck radius of the interparticle boundary (Figure 2).

The value of the initial particle boundary radius $a_{0}$, which refers to the state after the powder compaction (before sintering), can be calculated from the following relationships:

(12) $$a_{0}=\sqrt{\frac{r_{\min}u_{\mathrm{rn}}^{0}}{2}},$$

where $r_{\min}$ is the minimum radius of the particle contact and $u_{\mathrm{rn}}^{0}$ is the penetration of particles after compaction and before sintering. The evolution of the size of neck radius can be calculated by:

(13) $$\dot{a}=-\frac{r_{\min}v_{\mathrm{rn}}}{a}.$$

The maximum value of *a* indicating the end of sintering (the equilibrium state) can be described by the following geometric relationship:

(14) $$a_{\max}=r_{\min}\sin\frac{\Psi }{2}.$$

The detailed description of sintering model parameters is defined in Reference [20].

### 2.3. Cooling

Powder particle interaction during cooling is modeled using cohesive elastic model with damping.

## 3. Numerical Results and Discussion

The model described above was applied to the simulation of a hot pressing process for the mixture of 80% vol. intermetallic NiAl and 20% vol. ceramic Al${}_{2}$O${}_{3}$. The discrete element geometrical model of a representative mixture of NiAl and ceramic Al${}_{2}$O${}_{3}$ particles was generated. Simulations were performed using the parameters for single-phase contact interactions previously determined for pure NiAl and Al${}_{2}$O${}_{3}$ powders in Reference [20]. The parameters for two-phase mixed contact interaction were calibrated by performing numerical simulations of two-phase NiAl–20%Al${}_{2}$O${}_{3}$ powder and fitting the numerical density evolution of composite material to corresponding experimental data. Finally, the model was validated by numerical simulations of two-phase NiAl–20%Al${}_{2}$O${}_{3}$ powder performed at different process parameters.

### 3.1. Generation of the Specimen

The DEM geometrical model was generated by taking the particle size as the same as in the real powder and scaling down the powder specimen dimensions due to computational limitations. Such scaling is acceptable if the temperature and pressure distribution in the real specimen can be considered uniform.

**Figure 3:**
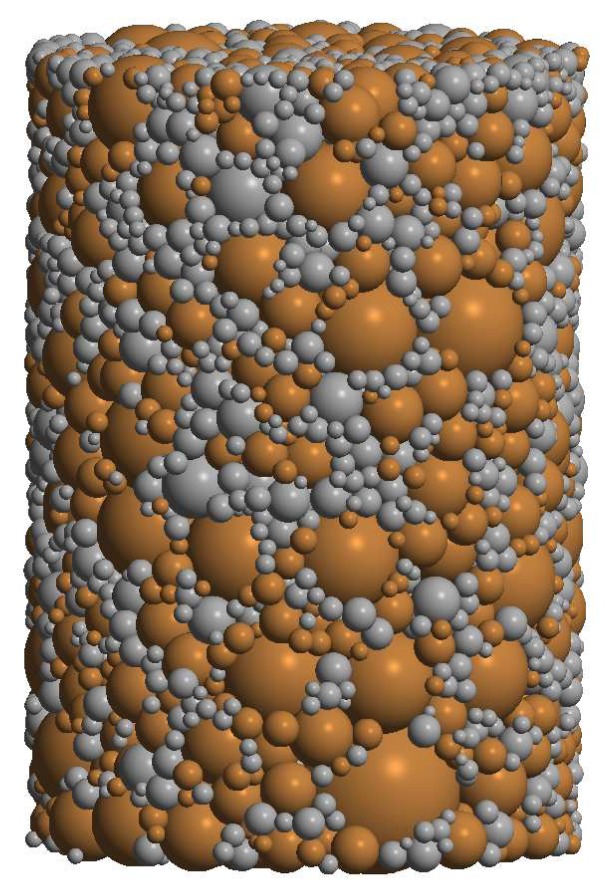
Discrete element specimen of two-phase 80%NiAl–20%Al${}_{2}$O${}_{3}$ powder.

The DEM specimen was generated using a specially designed procedure. The procedure consisted of the generation of randomly-distributed-in-space loose particles with sizes according to powder particle size distribution and the compaction of particles to achieve a densely packed specimen by a dynamic method (under prescribed contraction of the boundary surfaces). The algorithm of generation was described widely in Reference [20]. The generated specimen of 80%NiAl–20%Al${}_{2}$O${}_{3}$ composite materials is shown in Figure 3. The intermetallic phase is marked in dark orange, while the ceramic phase is grey. The statistical information of the particle size distributions in specimen is given in Table 1.

The DEM specimen generated with this algorithm satisfies the main requirements, such as material isotropy, uniform distribution of reinforcement (ceramic) particles in intermetallic NiAl matrix, irregular configuration of particles, real particle size distributions, relatively low porosity, and dense packing of powder particles.

The particle size distribution and size ratio of the two powders is an important factor in the sintering of composites [10]; therefore, the DEM specimen generated for simulations very closely reproduced the real particle size distribution of each phase in the powder mixture, which was given in the authors’ earlier work [29].

**Figure 4:**
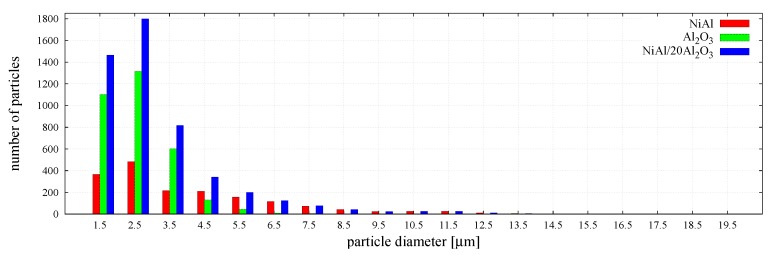
The distribution of the number of particle size of NiAl–20%Al${}_{2}$O${}_{3}$ powder mixture with its phases—NiAl and Al${}_{2}$O${}_{3}$.

**Figure 5:**
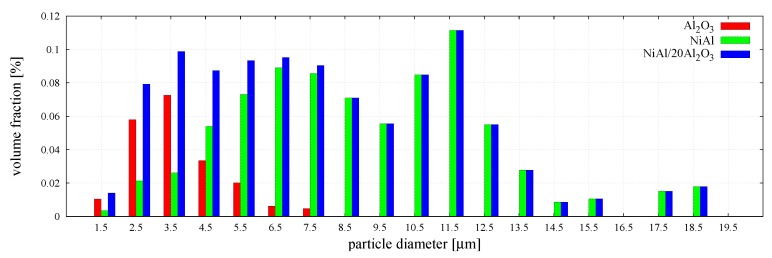
The distribution of the volume fraction of particle size of NiAl–20%Al${}_{2}$O${}_{3}$ powder mixture with its phases—NiAl and Al${}_{2}$O${}_{3}$.

The distributions of the number and the volume fractions of particles corresponding to each phase and the mixture are presented graphically in Figure 4 and Figure 5, respectively. The histograms in Figure 5 show that larger particles, despite their small number, occupy a significant volume fraction of the specimen.

### 3.2. Determination of the Model Parameters

**Figure 6:**
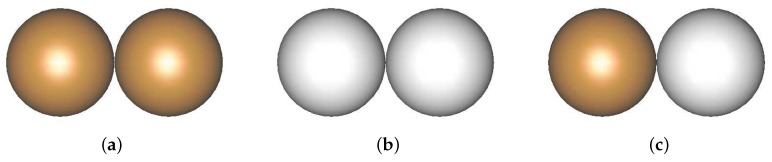
Three types of material interaction in the two-phase NiAl–Al${}_{2}$O${}_{3}$ powder: (**a**) NiAl–NiAl, (**b**) Al${}_{2}$O${}_{3}$–Al${}_{2}$O${}_{3}$, and (**c**) NiAl–Al${}_{2}$O${}_{3}$.

In the considered two-phase powder mixture, three types of material interaction: NiAl–NiAl, Al${}_{2}$O${}_{3}$–Al${}_{2}$O${}_{3}$, and NiAl–Al${}_{2}$O${}_{3}$, shown schematically in Figure 6, should be considered.

The material parameters of the sintering model for the NiAl–NiAl interaction via the simulation of pure NiAl powder were established in Reference [20]. The material parameters of the NiAl sintering model were estimated on the basis of equations and relations [20]. The estimation of alumina parameters was performed analogously. The material data of intermetallic and ceramic powders used in the calibration process are shown in Table 2.

Now, the model of sintering of the two-phase powder should be completed with the parameters for the NiAl–Al${}_{2}$O${}_{3}$ interaction. Beforehand, however, let us analyze the character of the NiAl–Al${}_{2}$O${}_{3}$ bond.

**Figure 7:**
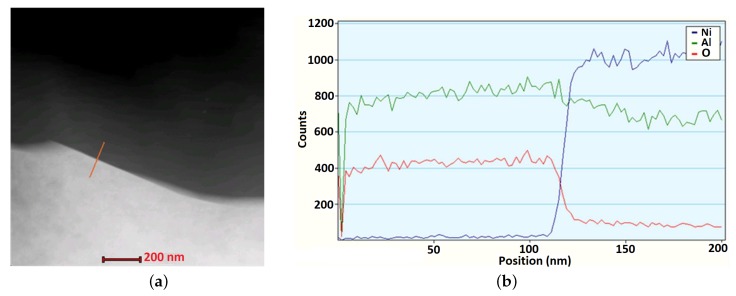
The micrograph of SEM (**a**) and the evolution of Al, O, and Ni as the content along the NiAl–Al${}_{2}$O${}_{3}$ interface (**b**) in the sample sintered in following conditions: $T_{s}=1400\;$C, $t_{s}=30$ min, p=30 MPa [30]. Thin lamellae of the NiAl–Al${}_{2}$O${}_{3}$ sample were cut using FIB Quanta 200 3D FEI instruments and thinned using a Leica EM RES 101 ion beam thinner.

The NiAl–Al${}_{2}$O${}_{3}$ interfaces in composites sintered at different process parameters were examined in the authors’ previous work [30]. The bonds between alumina and intermetallic particles, visible at some points, were initially observed in a sample sintered at temperature 1300 ${}^{\circ }C$; however, solid connections were only achieved with the rise of the sintering temperature. The interface was characterized by a good quality without the formation of any new phase, which may lead to a decrease in the main properties (e.g., mechanical) of the bond. The clean condition of the NiAl–Al${}_{2}$O${}_{3}$ interface was certified in the analysis accomplished using a TEM EDS detector. The evolution of the content of Ni, O, and Al elements was studied along the marked line across the interface (Figure 7). The TEM investigation confirmed that the connection between the intermetallic and ceramic particles was relatively strong and indicates an adhesive form without any presence of the phase transitions. Furthermore, the instant shift of contrast at the area of the interface also proved that no interface layer with a diffusive character was formed.

The adhesive character of the contact bond between the intermetallic and ceramic particles should be taken into account in the formulation of the discrete element model of sintering. The viscoelastic model, presented in Section 2, assumes the cohesion between interacting discrete elements and a good penetration of the particles resulting from the diffusive character of the contact bond. In the case of the NiAl–Al${}_{2}$O${}_{3}$ interface, the penetration of the particles depends mainly on the viscosity of a material and applied external pressure. Due to this fact, it has been assumed that the sintering driving force $F^{\mathrm{sint}}$, given by Equation (11), will be neglected in the model of interaction between the NiAl and Al${}_{2}$O${}_{3}$ particles during sintering.

The material model parameters for the contact interaction of NiAl and Al${}_{2}$O${}_{3}$ particles were estimated as follows: The mean atomic volume Ω was evaluated similarly to the method described in Reference [20], the grain boundary width δ the same as for the NiAl, and the effective Young’s modulus $\bar{E}$ was calculated through Equation (2).

The material data of mixed contact interaction used in the calibration process are shown in Table 3. The diffusive parameters (the pre-exponential factor of the grain boundary diffusion $D_{0\mathrm{gb}}$ and the activation enthalpy of grain boundary diffusion $\Delta H_{\mathrm{gb}}$) will be employed as the fitting parameters in a similar way as in the case of calibration of the sintering model of one-phase powder. The material parameters of the sintering model of the NiAl–Al${}_{2}$O${}_{3}$ interaction, evaluated in the way described above and tuned in the calibration procedure, are shown in Table 3.

### 3.3. Simulation Results

**Figure 8:**
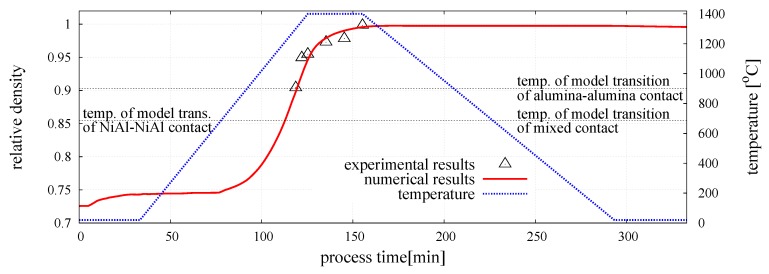
Evolution of the relative density of the mixture of NiAl–Al${}_{2}$O${}_{3}$ powder.

The numerical model of composite was calibrated for the following sintering process parameters: External pressure p=30 MPa, sintering temperature $T_{s}=1400{\;}^{\circ }C$ (1673 K), and sintering time $t_{s}=30$ min. The numerical and experimental evolution of the relative density of the sintered NiAl–20%Al${}_{2}$O${}_{3}$ specimen is plotted in Figure 8, together with the temperature profile. The relative density of the composite was evaluated from:

(15) $$\rho_{\mathrm{rel}}=\frac{\rho }{\rho_{\mathrm{theo}}},$$

where ρ is the bulk density and $\rho_{\mathrm{theo}}$ is the temperature-dependent theoretical density of the composite given by the following relation:

(16) $$\rho_{\mathrm{theo}}=\frac{V_{\mathrm{mat}}\rho_{\mathrm{theo}}^{0,m}}{{\left(1+\alpha_{\mathrm{mat}}\Delta T\right)}^{3}}+\frac{V_{\mathrm{reinf}}\rho_{\mathrm{theo}}^{0,r}}{{\left(1+\alpha_{\mathrm{reinf}}\Delta T\right)}^{3}},$$

where $\rho_{\mathrm{theo}}^{0,m}$ and $\rho_{\mathrm{theo}}^{0,r}$ are the theoretical densities of the composite matrix (NiAl) and reinforcement (Al${}_{2}$O${}_{3}$) at room temperature, $\alpha_{\mathrm{mat}}$ and $\alpha_{\mathrm{reinf}}$ are the linear thermal expansion coefficients of the matrix and reinforcement, $V_{\mathrm{mat}}$ and $V_{\mathrm{reinf}}$ are the volume fractions of each phase, and ΔT is the temperature increment. The values of the theoretical densities and the linear coefficients of the thermal expansion of NiAl and alumina materials are given in Table 2.

The numerical results were compared with our own experimental results presented in Reference [29]. Figure 8 shows a very good agreement of the numerical and experimental results, which confirms that the material parameters of the sintering model have been determined properly. The character of the curve representing the evolution of the relative density of the composite obtained in the simulation is similar to the evolution curves for the pure intermetallic presented in Reference [20]. Similarly, an initial compaction related to the application of the external pressure of 30 MPa can be observed. The value of the relative density does not change until the temperature of the sintering activation of intermetallic particles is achieved (T=683C). At this point, the sintering model is activated. In the initial stage of sintering, the densification rate is quite low, but a gradual growth of temperature and the activation of sintering of ceramic particles (T=899C) accelerated the densification process.

**Figure 9:**
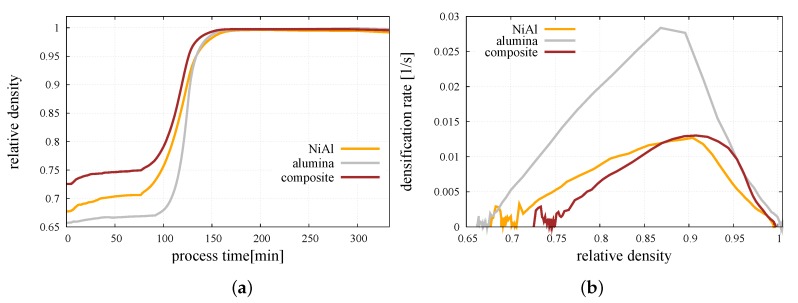
Comparison of: (**a**) Relative density evolution, (**b**) densification rate of pure NiAl, pure alumina, and mixture of NiAl/Al${}_{2}$O${}_{3}$ powder sintered in $T_{s}=1400{\;}^{\circ }$C and p=30 MPa.

Figure 9a presents a comparison of the relative density evolution of the pure NiAl, pure alumina, and mixture of the NiAl/Al${}_{2}$O${}_{3}$ powders sintered in $T_{s}=1400{\;}^{\circ }$C and p=30 MPa. The sintering of the intermetallic powder is characterized by a long densification with a relatively low rate, while alumina achieve a high relative density in a short time with a high rate. A comparison of the densification rate as a function of the relative density of all the studied powders is made in Figure 9b.

It has to be mentioned that the composite material started the sintering stage from the highest relative density due to relatively high diversity and a privileged distribution of the particle size, which reduces the porosity. Pure powders started the whole process from a lower relative density, and only the intermetallic powder has been compacted during compression due to a relatively low Young’s modulus. The initial fluctuations of densification rate (Figure 9b) occuring at the compaction stage are related to the reduced size of the numerical sample and the specific character of the densification process at the initial stage of hot pressing (Figure 9a) resulting from micromechanical effects, such as particle rearrangement [24]. Since the densification rate is calculated as the increment of relative density in a certain increment of time [20], its character depends strongly on the smoothness of the relative density curve.

As in the case of pure powders, the composite material indicates the highest densification rate at the intermediate stage of sintering and gradually slows down after reaching the sintering temperature. While the sintering stage was finished, the relative density of composite material achieved a value very close to 1, which refers to the obtainment of almost fully dense material.

**Figure 10:**
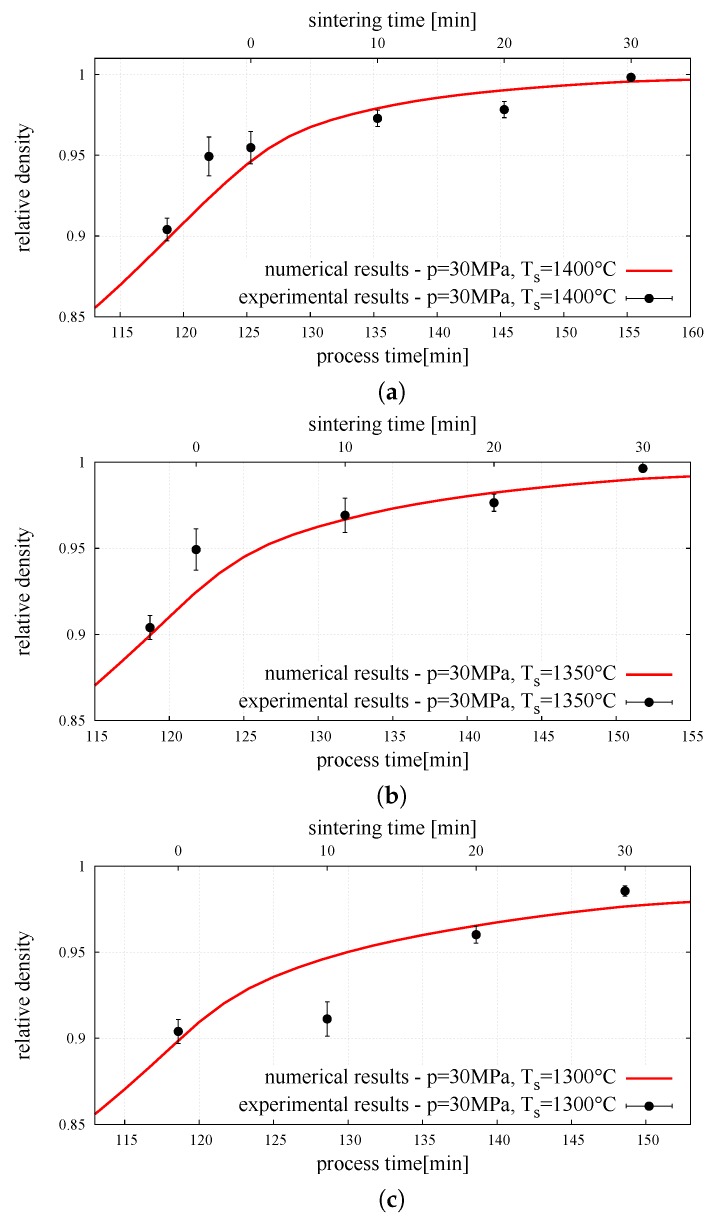
Density evolution—numerical and experimental results (with standard deviation error bars) [29] of composite NiAl–Al${}_{2}$O${}_{3}$ powder sintering for pressure 30 MPa and sintering temperature of: (**a**) $T_{s}=1400{\;}^{\circ }C$, (**b**) $T_{s}=1350{\;}^{\circ }C$, (**c**) $T_{s}=1300{\;}^{\circ }C$.

Finally, the DEM model of powder metallurgy of two-phase material was employed in the validation procedure by comparing numerical results with our own experimental ones presented in Reference [29]. For validation purposes, a comparison was made for for sintering temperature $T_{s}$, different than that assumed for calibration. The sintering time was the same for all the analyses ($t_{s}=30$ min). The relative density evolution obtained in the numerical simulation was compared with experimental measurements in Figure 10. The graph shows the sintering stage only, excluding the compaction and cooling stages. The comparison of the numerical and an experimental results of composite relative density brought a positive conclusion. The numerical representations of all composite sintering processes with different temperature and time are nearly identical to experiments results—numerical lines highly cover the experimental density points, showing a really good correspondence.

**Figure 11:**
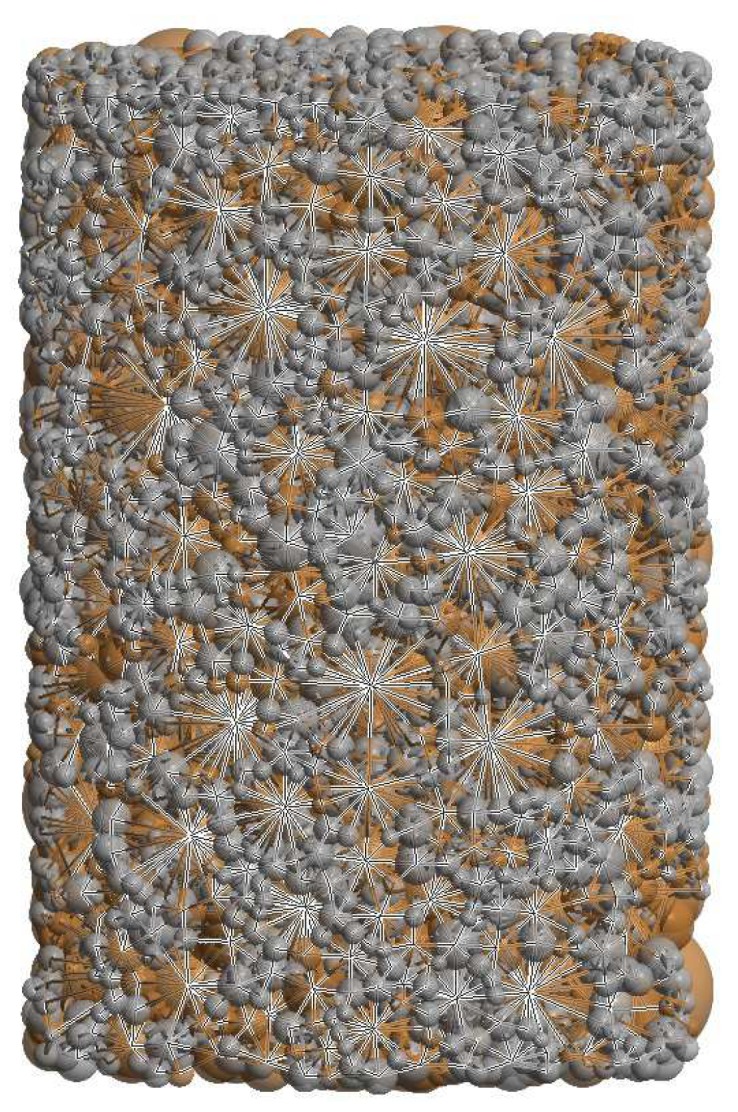
Network of particle connections during sintering.

The macroscopic behaviour of the mixture of two-phase powder during the sintering process has its source at the microscopic scale. All of the changes of specimen densification occurring during the compaction, sintering or heating, resulted from the sum of many interactions between powder particles. Figure 11 presents the composite specimen during hot pressing with the network of particle interactions represented by beams connecting the centers of interacting particles.

**Figure 12:**
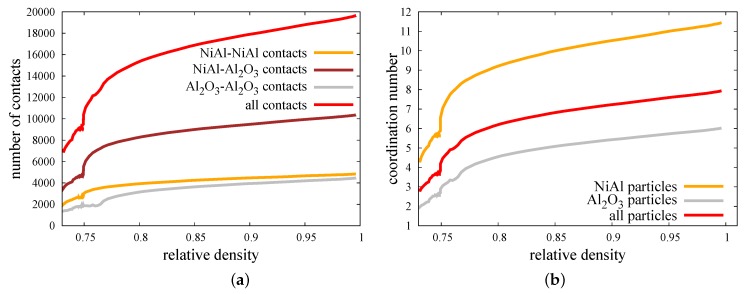
Evolution of (**a**) the number of contacts, (**b**) the coordination number of composite NiAl–Al${}_{2}$O${}_{3}$ specimen during hot pressing process.

In the case of the two-phase powder sample, where the three types of material contacts can be found (Figure 6), the discrete element analysis allows to split all interaction into the certain sorts and study them separately. On this basis, Figure 12 presents the evolution of the number of contacts of each material type and the coordination number of each particle phase of the NiAl–Al${}_{2}$O${}_{3}$ specimen during the hot pressing process for pressure 30 MPa and sintering temperature of $T_{s}=1400{\;}^{\circ }C$. The coordination number $n_{c}$ is defined as the average number of contacts per particle, and it was calculated as follows:

(17) $$n_{c}=\frac{2N_{c}}{N_{p}},$$

where $N_{c}$ is the total number of contacts between the $N_{p}$ particles in the specimen.

In order to evaluate the coordination number of each phase (matrix or reinforcement particles) $n_{c}^{m,r}$, Equation (17) should be rewritten as follows:

(18) $$n_{c}^{m,r}=\frac{2N_{c}^{m-m,r-r}+N_{c}^{m-r}}{N_{p}^{m,r}},$$

where $N_{c}^{m-m,r-r}$ is the number of contacts between the particles of each phase (matrix–matrix or reinforcement–reinforcement) and $N_{p}^{m,r}$ is the number of particles of each phase in the specimen.

The micromechanical results presented in Figure 12 are in agreement with a macroscopic quantities—evolution of relative density (Figure 8) and densification rate (Figure 9). The beginning of a hot pressing process consisting of the application of external pressure is accompanied by the increase of a number of contacts of all material types. This issue is also reflected in the increase of the coordination number. At this point of densification level, the mixed contacts are around 50% of all contacts presented in the composite specimen. The coordination number of NiAl particles is around 2.5 times higher than the coordination number of ceramic particles, which can be explained by the bigger size of intermetallic particles and their surfaces.

Reaching the temperature of sintering activation between intermetallic particles and between intermetallic–ceramic particles (mixed contact) indicates the further dynamic growth of a number of contacts, especially in the case of mixed contact. A delay of this phenomenon for ceramic–ceramic material contact (at 0.77 of relative density) is associated with the higher temperature of sintering activation for this pair. Despite this fact, the coordination number of Al${}_{2}$O${}_{3}$ particles rises at 0.75 of relative density because of the growth of a number of a mixed material contact. The total number of contacts during sintering (from the beginning at 0.75 of relative density to achieving full density) increases around two times for all contacts, 1.76 times for the NiAl–NiAl contact, 2.16 times for the mixed contact, and 2.32 times for ceramic–ceramic contact. The highest growth of coordination number during the sintering can be seen for NiAl particles—from around six up to 11.5.

**Figure 13:**
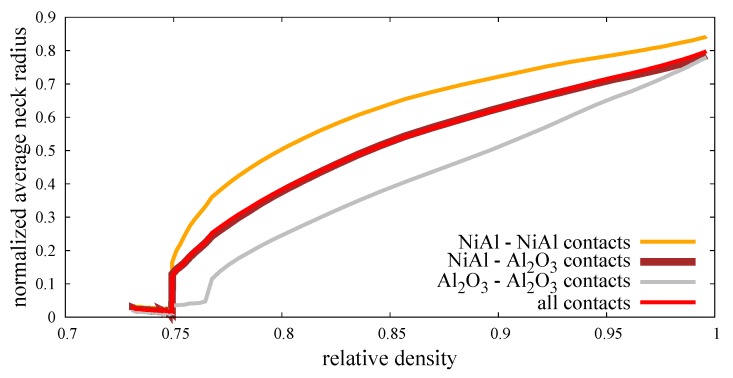
Evolution of the normalized average neck radius.

**Figure 14:**
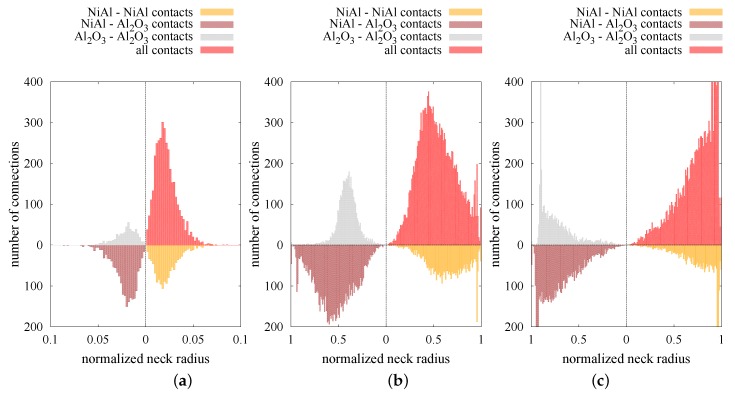
Distribution of normalized neck radii of composite NiAl–Al${}_{2}$O${}_{3}$: (**a**) After loading ($\rho_{\mathrm{rel}}$ = 0.75), (**b**) in the intermediate stage of sintering ($\rho_{\mathrm{rel}}$ = 0.87), and (**c**) after sintering ($\rho_{\mathrm{rel}}$ = 0.995).

The increase in the number of contacts and, hence, the coordination number results from the high mobility of particles due to the application of external pressure and sintering driving force. Moreover, the high temperature affects the viscosity of particles at a relatively low level, which allows the particles to overlap. The penetration of particles has a great impact on the size of the neck radius (Equations (12) and (13)), which can be treated as the parameter determining the densification level at the microscale. The micro–macro dependence of parameters indicating the densification level (normalized average neck radius $a/r_{\min}$ vs. relative density $\rho_{\mathrm{rel}}$) is shown in Figure 13. The calculated average neck radius was divided by the corresponding minimal radius of each contact pair, as in Reference [22]. As in Figure 12a, the normalized average neck radius was split into the interaction of the certain material types. In the same way, the distributions of normalized average neck radii of the composite specimen at three particular moments of the process (after loading, at the intermediate stage of sintering, after sintering) in the form of histograms are presented in Figure 14. The three types of material contact together with all contacts are presented in each quarter in Figure 14.

The results confirm that a low state of densification (after compression, before sintering) is accompanied by a large number of small neck radii of particle contacts. The normalized average neck radius equals approximately 0.02, regardless of the connection type (Figure 13). This effect can also be seen in Figure 14a, where the peak (maximum number of connections at the certain size) lies around a 0.02 value. As sintering begins, the normalized average neck radius increases and its distribution evolves (Figure 14b). Moreover, the distributions of each material contacts show various shapes. The NiAl–NiAl type becomes relatively wide with nearly none of the connections with a small size and many of the big ones (from 0.5 to 0.8). The lack of small size bonds can be explained by the low number of new intermetallic connections at the intermediate and final stage of sintering (Figure 12a). The mixed type shows a higher peak, indicating the specific averaged value (Figure 13) of $a/r_{\min}\approx$ 0.5. The shape of the histogram is pretty broad, ranging from 0.1 to 0.9 of a normalized neck radius. The distribution of a ceramic material type differs mostly from the intermetallic one. Most of the connections oscillate around 0.4 and the shape of histogram is quite narrow.

As can be expected, the final stage of sintering ($\rho_{\mathrm{rel}}$ closed to 1) brings the distributions with a large number of connections with big sizes of $a/r_{\min}$; however, there are still connections with the potential for further sintering (penetration). The reason for the lower mobility of the particles, which reflects in a lower densification rate at the final stage of sintering, is related to the higher viscosity of particles blocking further particle penetration. Furthermore, a subsequent decrease in densification refers to the gradual end of sintering of each discrete element contact pair and its transition to an equilibrium state.

**Figure 15:**
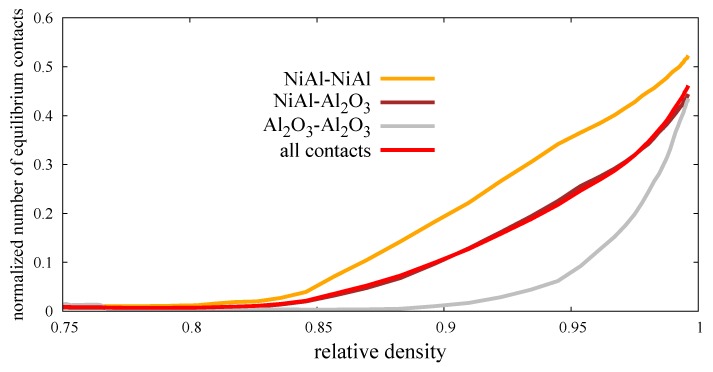
Evolution of the normalized number of equilibrium contacts of composite NiAl–Al${}_{2}$O${}_{3}$ specimen during the hot pressing process.

Figure 15 presents the normalized number of equilibrium contacts (ratio of the number of equilibrium contacts and the total number of contacts) in the function of relative density. In other words, the graph shows how many contacts (with respect to all contacts) have been shifted into the cohesive elastic contacts, with damping indicating the end of sintering after they achieve the maximum size of neck radius $a_{\max}$ (Equation (14)). Here, the relation of $a_{\max}$ depends on the minimum radius of particle contact. In many papers [15,16], the following parameter depends on the effective radius, which can lead to some inconsistencies. In the case of significant difference between the size of particles, the value of $a_{\max}$ will be much bigger than the radius of lower particles, which is a rather nonphysical and unacceptable issue.

The presented normalized number of equilibrium contacts at the relative density closed to 1 is rather equal for each type of material contacts (around 0.5); however, their evolution differs. The growth of a number of equilibrium contacts of intermetallic pairs indicates the linear character with a constant rate, while the ceramics pairs are characterized by the nonlinear one. The high rate of the transition from the sintering to cohesive contact of ceramic pairs is related to the value of the dihedral angle (Table 2), which was set by the calibration procedure of pure alumina powder (Section 3.2). As the dihedral angle is lower, the equilibrium state is set earlier (at the lower value of $a_{\max}$) which is proven by comparing the NiAl–NiAl and Al${}_{2}$O${}_{3}$–Al${}_{2}$O${}_{3}$ curves.

## 4. Conclusions

An original numerical model of the powder metallurgy process of a two-phase powder mixture was formulated and implemented within the discrete element framework. All the stages of the hot pressing, including initial powder compaction, heating, sintering, unloading, and cooling, can be analyzed efficiently. The DEM model of a two-phase system requires a characterization of the interactions between the particle pairs of the same and different materials. The parameters for the interaction of NiAl–NiAl and Al${}_{2}$O${}_{3}$–Al${}_{2}$O${}_{3}$ particle pairs were determined using experimental results for the hot pressing of respective single phase powders. Based on the results of microscopic studies of the NiAl–Al${}_{2}$O${}_{3}$ interface, a special model for the adhesive NiAl–Al${}_{2}$O${}_{3}$ interaction was proposed. Its parameters were determined in the calibration procedure.

In order to numerically represent the sintered sample as best as possible, the special algorithm of the generation of the discrete element geometrical model was developed. The generated DEM geometrical models satisfy all the main requirements of a real two-phase powder body after mixing, such as the isotropy of composite material or the real distribution and size of reinforcement (ceramic) particles in an intermetallic NiAl matrix. Satisfactory results of calibration and validation prove a good performance of the developed numerical model. The discrete element modeling of powder metallurgy process has allowed us to obtain a numerical representation of sintered specimens for various combinations of the sintering process parameters.

The evolution of macroscopic quantities was confirmed through micromechanical analysis. Parameters, such as the number of contacts, coordination number, and neck size distributions, were studied with respect to process time. The investigation at a microscopic level allows to study the micromechanical parameters with respect to material type contacts. The results from the micromechanical analysis are in agreement with changes in relative density and the densification rate of the sintered composite.

## Figures and Tables

**Table 1:** Statistical parameters of the particle size distribution in the specimen [μm].

Statistical Parameter/Material	NiAl Fraction	Al${}_{2}$O${}_{3}$ Fraction	NiAl–20%Al${}_{2}$O${}_{3}$ Mixture
Mean value	3.97	2.48	3.01
Standard deviation	2.50	0.89	1.80
Maximum value	18.61	5.88	18.61
Minimum value	1.50	1.25	1.25
Number of particles	1751	3201	4952

**Table 2:** Materials model parameters of contact interaction between NiAl–NiAl and Al${}_{2}$O${}_{3}$–Al${}_{2}$O${}_{3}$ particles.

Material Constant	Parameter Value	
	NiAl–NiAl	Al${}_{2}$O${}_{3}$–Al${}_{2}$O${}_{3}$
Mean atomic volume, Ω [m${}^{3}$]	$1.20\times 10^{-29}$	$8.47\times 10^{-30}$
Pre-exponential factor of the grain boundary diffusion, $D_{0\mathrm{gb}}$ [m${}^{2}/$s]	$2.55\times 10^{-5}$	9.751
Activation enthalpy of grain boundary diffusion, $\Delta H_{\mathrm{gb}}$ [kJ/mol]	185	389
Grain boundary width, δ [nm]	0.5	0.5
Young’s modulus, *E* [GPa]	183	404
Poisson’s ratio, ν	0.34	0.232
Surface energy, $\gamma_{s}$ [J/m${}^{2}$]	1.57	1.28
Dihedral angle, Ψ [${}^{\circ }$]	147	127
Density, $\rho_{\mathrm{theo}}$ [kg/m${}^{3}$]	5910	3970
Coefficient of thermal expansion, α [$10^{-6}$ K${}^{-1}$]	11.5	7.4

**Table 3:** Materials model parameters of contact interaction between NiAl and Al${}_{2}$O${}_{3}$ particles.

Material Constant	Parameter Value
Mean atomic volume, Ω [m${}^{3}$]	$9.01\times 10^{-30}$
Pre-exponential factor of the grain boundary diffusion, $D_{0\mathrm{gb}}$ [m${}^{2}/$s]	$3\times 10^{-2}$
Activation enthalpy of grain boundary diffusion, $\Delta H_{\mathrm{gb}}$ [kJ/mol]	280
Grain boundary width, δ [nm]	0.5
Effective Young’s modulus, $\bar{E}$ [GPa]	279
